# Treatment of hereditary angioedema with recombinant human C1 Inhibitor in a real-life setting: the experience of the HAE Centre in Milan

**DOI:** 10.1186/1939-4551-8-S1-A173

**Published:** 2015-04-08

**Authors:** Andrea Zanichelli, Maddalena Wu, Marco Cicardi, Romualdo Vacchini, Erika Bonanni

**Affiliations:** 1 Università Di Milano, Ospedale Luigi Sacco, Italy

## Background

Hereditary angioedema due to C1 inhibitor deficiency (C1-INH-HAE) is a rare disease characterized by recurrent episodes of cutaneous, abdominal and laryngeal edema. Bradykinin is the mediator of increased vascular permeability and edema formation. Treatment of HAE attacks in Italy is based on the administration of human C1 inhibitor or bradykin receptor antagonist. A recombinant human C1 inhibitor (rhC1INH) is marketed in Italy since 2012 for the treatment of HAE in adults. Safety and efficacy of rhC1INH were documented in several phase III trials. This analysis reports characteristics and treatment outcomes of HAE attacks treated with rhC1INH in a real-life setting.

## Methods

Patients diagnosed with C1-INH-HAE, based on clinical manifestations of angioedema and on laboratory tests confirming C1-INH deficiency, prescribed with rhC1INH, were included in this analysis. Before rhC1INH administration patients were tested for anti-rabbit epithelium IgE. Time to treatment (time from onset of symptoms to drug administration), initial relief of symptoms within 4 hours and time to resolution (time from drug administration to complete symptoms resolution) were recorded. Data on adverse events were also collected.

## Results

12 patients (7 females; median age 39 years) with HAE were prescribed with rhC1INH. Anti-rabbit epithelium IgE were negative in all patients. RhC1INH was administered for the treatment of 33 HAE attacks: 22 cutaneous attacks, 8 abdominal, 1 laryngeal and 2 involving multiple locations (laryngeal and cutaneous, abdominal and cutaneous, respectively). Median time to treatment was 2 hours. Initial relief within 4 hours was achieved in 93% of attacks. Median time to resolution was 10 hours. In 2 abdominal attacks, rhC1INH was used in a lower dose than recommended and initial symptoms relief was not achieved within 4 hours. In 1 of those two attacks, a second treatment with rhC1INH was administered after 30 hours from the first treatment with subsequent symptoms resolution in 6 hours. In the other attack, no additional infusion with rhC1INH was administered and attack resolution was achieved in 24 hours. Adverse events registered after treatment with rh-C1-INH were: headache (15% of attacks), erythema (12% of attacks), tingling (12% of attacks).

## Conclusions

The treatment with rhC1INH in real life-setting was effective in reverting angioedema symptoms in all location. In 2 attacks treated with a lower dose than the approved 50U/kg, symptoms resolution was slower and in one case a second infusion was needed. No serious adverse events related to drug administration were recorded.

